# Fatal Cat Scratch Disease in Alcoholic Patient With Liver Cirrhosis: A Case Report

**DOI:** 10.7759/cureus.60609

**Published:** 2024-05-19

**Authors:** Lital Nainshtein-Baturov, Eleonora Achrak, Yisroel Y Grabie, Kalim Khan

**Affiliations:** 1 Department of Osteopathic Medicine, Touro College of Osteopathic Medicine, New York, USA; 2 Department of Internal Medicine, Northwell Health - Staten Island University Hospital, New York, USA

**Keywords:** mortality, infection, cat scratch, sepsis, immunocompromised, alcoholic liver cirrhosis

## Abstract

Cat-scratch disease (CSD), a human infection resulting from *Bartonella* species, commonly manifests as tender lymphadenopathy. Consequently, its inclusion in the differential diagnosis of fevers of unknown origin and lymphadenopathy syndromes is imperative. Typically, it manifests as self-limiting tender lymphadenopathy and does not lead to fatalities, though it may assume a more severe course in immunocompromised individuals. Diagnostic challenges often surround CSD due to its elusive nature in laboratory tests, necessitating a reliance on the clinical presentation for definitive diagnosis. This can manifest in delayed procedures and testing, which can prolong intervention and cause rapid progress of bacteria, potentially causing severe complications and death. In this case report of a 58-year-old Caucasian male, we delve into the clinical presentation and eventual fatality of CSD in a patient with liver cirrhosis, occurring in the United States. He sought care in the emergency department due to lethargy, fever, and swollen axilla following a cat scratch. Although the patient did not exhibit signs of sepsis upon admission, he rapidly progressed to sepsis and passed away within 24 hours. This case highlights the significance of timely and proactive management in individuals presenting with CSD, especially when complicated by underlying immunocompromised conditions. Early recognition, the administration of suitable antibiotics, and comprehensive supportive care are pivotal in averting fatal outcomes in such cases.

## Introduction

Cat-scratch disease (CSD), primarily caused by *Bartonella henselae* (*B. henselae*), a fastidious gram-negative bacilli bacterium [1] presents a diagnostic challenge due to its non-specific symptoms, complicating swift clinical identification. The carriers for *B. henselae* are asymptomatic cats, which contract the bacteria from common cat fleas, Ctenocephalides felis [2]. These fleas reside in the gut and are expelled onto the cat's skin via flea dropping. When cats groom themselves, they cause the bacteria from their skin to further spread to their mouths and claws. Therefore, humans can get infected from a bite or scratch of an infected cat, causing the transfer of flea feces into an open wound. Upon entering the human body, *B. henselae* infects erythrocytes and endothelial cells, leading to a prolonged intraerythrocytic viremia and facilitating systemic dissemination across the host. This bacterium has evolved several mechanisms to evade the human immune system, which includes the inhibition of apoptosis in infected cells and avoiding recognition by phagocytes through its unique outer membrane proteins.

Clinical manifestations of CSD can include a wide range of non-specific symptoms and its benign course in immunocompetent individuals; it can be easily overlooked, delaying a diagnosis. The traditional diagnostic criteria of CSD include (1) cat contact and a scratch or inoculation event history, (2) positive cat scratch skin test, (3) regional lymphadenopathy without apparent cause, and (4) specific histopathologic features on biopsy. Furthermore, polymerase chain reaction (PCR)-based assays have identified cases lacking these traditional criteria [3]. Keeping these criteria in mind while considering clinical presentation is essential for the accurate identification and timely management of CSD this disease.

In patients with alcoholic liver cirrhosis, the pathophysiological course of CSD takes a more severe trajectory, potentially leading to systemic complications such as bacteremia, endocarditis, and neurologic involvement. Liver cirrhosis is a significant medical condition, often resulting from chronic alcohol consumption. It is characterized by extensive liver tissue damage, fibrosis, and nodular regeneration, which ultimately impairs hepatic function. Chronic alcohol abuse has been identified as a leading cause of liver cirrhosis, exacerbating susceptibility to infections due to its immunosuppressive effects. This heightened susceptibility extends to CSD, where the compromised immune system may facilitate more aggressive disease presentations. The management of CSD in immunocompromised individuals necessitates vigilant care, including rapid diagnosis, appropriate antibiotic therapy, and close monitoring, to mitigate potentially devastating outcomes [4]. In this case report, we show a clinical scenario that highlights the interplay between alcoholic liver cirrhosis, immunosuppressive states, and CSD. By elucidating the recent pathophysiological mechanisms at play and the clinical consequences of these interactions, we aim to contribute to a deeper understanding of the challenges posed by infections in this vulnerable patient population.

## Case presentation

A 58-year-old Caucasian male with a history of alcoholic liver cirrhosis, ethanol use disorder, chronic obstructive pulmonary disease (COPD), coronary artery disease (CAD), and heart failure with mid-range ejection fraction (HFmrEF) was admitted to the emergency department presenting with lethargy, stiff neck, and fever. This presentation followed a cat scratch on his right dorsal forearm, inflicted by his pet cat, that occurred approximately three days before his admission. On arrival, the patient was tachycardic to 130 bpm, hypotensive to 90/59, and fever of 100 F. This prompted immediate fluid resuscitation with 500 cc of lactated Ringer's solution and the initiation of empiric antibiotic therapy with vancomycin and cefepime to cover a broad spectrum of pathogens, including those specific to CSD. Although the patient initially responded to fluid resuscitation, demonstrating a transient improvement in blood pressure, he experienced a recurrence of hypotension shortly thereafter. Upon physical examination, the patient appeared lethargic, with full range of motion in his neck but with some complaints of stiffness on flexion and extension without significant pain. Additionally, a small 5 cm abrasion was noted over the dorsal right forearm, with no associated edema, fluctuance, drainage, or erythema. Mild edema was observed in the right axilla, with no overlying skin changes and no palpable lymphadenopathy.

**Figure 1:**
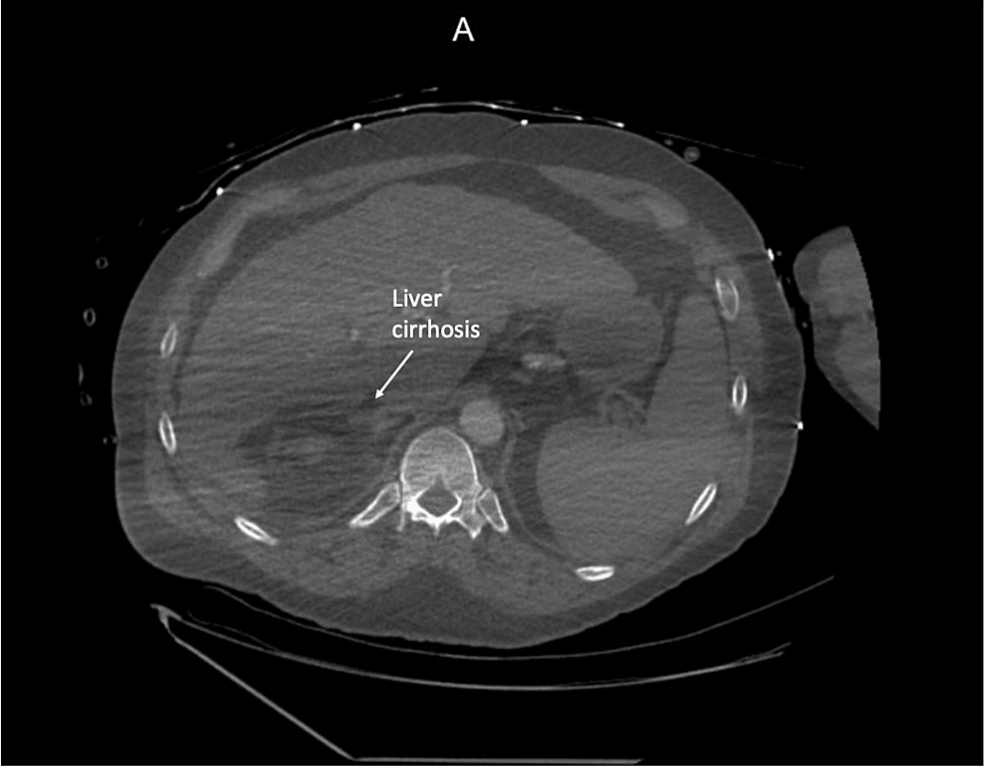
CT scan of the abdomen and pelvis shows signs of liver cirrhosis with portal hypertension

A CT scan of the abdomen and pelvis without contrast performed shortly after admission revealed signs of liver cirrhosis with portal hypertension, as shown in Figure 1*.*

**Figure 2:**
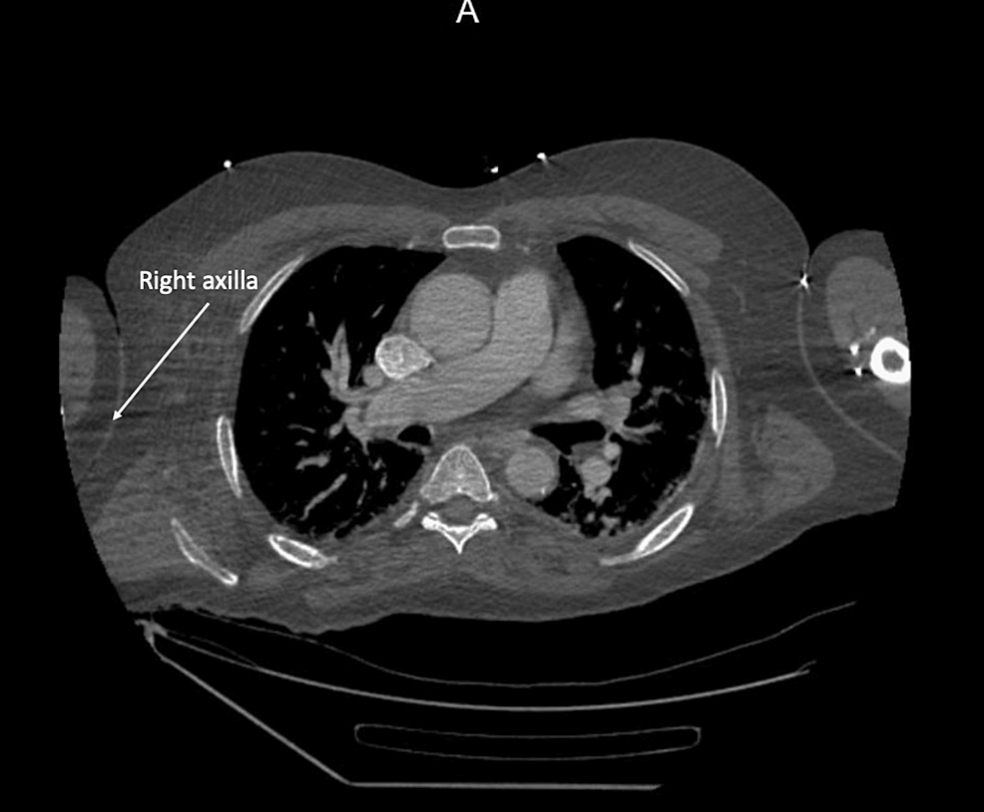
CT scan of the abdomen and pelvis shows nonspecific fat stranding in the right axilla, without evidence of focal fluid collection or subcutaneous gas

CT scan of the abdomen and pelvis also revealed nonspecific fat stranding in the right axilla, without evidence of focal fluid collection or subcutaneous gas (Figure 2).

Despite these interventions, the patient's condition rapidly deteriorated over 24 hours. He developed increased lethargy and hypoxia, requiring bilevel positive airway pressure (BiPAP) support due to desaturations down to 80% on room air. Initial laboratory findings in the emergency department showed leukocytosis with a white blood cell count of 24 x10^9/L, elevated bilirubin levels at 8.2 mg/dL (direct bilirubin 6.4 mg/dL), creatinine at 3.9 mg/dL (baseline 0.7 mg/dL), and an international normalized ratio (INR) of 2.2, indicating an acute exacerbation of his chronic liver disease, possibly acute kidney injury (hepatorental syndrome as a complication of his cirrhosis), and the development of sepsis (Table 1).

**Table 1:** Blood test indications of worsening chronic liver condition and potential sepsis ALT (SGPT): alanine aminotransferase (serum glutamic-pyruvic transaminase), AST (SGOT): aspartate aminotransferase (serum glutamic-oxaloacetic transaminase), eGFR: estimated glomerular filtration rate

Test	Result	Reference range
Complete blood count (CBC)		
WBC count	28.34	4.0-11.0 x10^3/µL
RBC count	4.22	4.20-5.70 x10^6/µL
Hemoglobin	15.0	13.5-17.5 g/dL
Hematocrit	44.5%	38.8-50.0%
Mean cell volume (MCV)	105.5	80-96 fL
Mean cell hemoglobin (MCH)	35.5	27-33 pg
Mean cell hemoglobin concentration (MCHC)	33.7	32-36 g/dL
Red cell distribution width (RDW)	15.3%	11.5-14.5%
Platelet count	78	150-400 x10^3/µL
Mean platelet volume (MPV)	11.6	7.4-10.4 fL
Differential		
Neutrophils absolute	21.17	1.8-7.7 x10^3/µL
Lymphocytes absolute	0.99	1.0-4.0 x10^3/µL
Monocytes absolute	3.46	0.2-0.8 x10^3/µL
Eosinophils absolute	0.00	0.0-0.4 x10^3/µL
Basophils absolute	0.00	0.0-0.2 x10^3/µL
Coagulation		
Prothrombin time (PT)	25.70	11.0-13.5 seconds
International normalized ratio (INR)	2.20	0.8-1.1
Activated partial thromboplastin time (aPTT)	64.0	25-35 seconds
Chemistry		
Sodium	138	136-145 mmol/L
Potassium	4.4	3.5-5.1 mmol/L
Chloride	97	98-107 mmol/L
Carbon dioxide	17	22-29 mmol/L
Anion gap	24	3-11 mmol/L
Blood urea nitrogen (BUN)	49	8-21 mg/dL
Creatinine	3.9	0.7-1.3 mg/dL
Glucose	98	70-99 mg/dL
Liver panel		
Albumin	3.2	3.5-5.0 g/dL
Total bilirubin	8.2	0.1-1.2 mg/dL
Alkaline phosphatase	130	40-129 U/L
AST (SGOT)	68	10-40 U/L
ALT (SGPT)	30	7-56 U/L
eGFR	17	>60 mL/min/1.73m^2

The patient was admitted to the intensive care unit where blood cultures and a second set of blood work were drawn. The time duration between the first and second sets of blood work was 17 hours. The blood cultures were negative and new blood work showed persistent leukocytosis of 24 x10^9/L, lactate of 14 mmol/L, potassium 6.2 mmol/L, carbon dioxide 13 mmol/L, blood urea nitrogen 53 mg/dL, creatinine of 4.8 mg/dL and albumin of 1.9 g/dL as shown in Table 2. These results indicate worsening conditions and progression of his acute kidney injury.

**Table 2:** Second set of blood work ALT (SGPT): alanine aminotransferase (serum glutamic-pyruvic transaminase), AST (SGOT): aspartate aminotransferase (serum glutamic-oxaloacetic transaminase), eGFR: estimated glomerular filtration rate

Test	Result	Reference range
Complete blood count (CBC)		
WBC count	24.0	4.0-11.0 x10^3/µL
RBC count	3.38	4.20-5.70 x10^6/µL
Hemoglobin	12.0	13.5-17.5 g/dL
Hematocrit	38.9%	38.8-50.0%
Mean cell volume (MCV)	115.1	80-96 fL
Mean cell hemoglobin (MCH)	35.5	27-33 pg
Mean cell hemoglobin concentration (MCHC)	30.8	32-36 g/dL
Red cell distribution width (RDW)	15.9%	11.5-14.5%
Platelet count	91	150-400 x10^3/µL
Mean platelet volume (MPV)	11.9	7.4-10.4 fL
Differential		
Neutrophils absolute	16.87	1.8-7.7 x10^3/µL
Lymphocytes absolute	4.38	1.0-4.0 x10^3/µL
Monocytes absolute	2.40	0.2-0.8 x10^3/µL
Eosinophils absolute	0.05	0.0-0.4 x10^3/µL
Basophils absolute	0.03	0.0-0.2 x10^3/µL
Coagulation		
Prothrombin time (PT)	26.80	11.0-13.5 seconds
International normalized ratio (INR)	2.29	0.8-1.1
Activated partial thromboplastin time (aPTT)	66.7	25-35 seconds
Chemistry		
Sodium	144	136-145 mmol/L
Potassium	6.2	3.5-5.1 mmol/L
Chloride	104	98-107 mmol/L
Carbon dioxide	13	22-29 mmol/L
Anion gap	27	3-11 mmol/L
Blood urea nitrogen (BUN)	53	8-21 mg/dL
Creatinine	3.9	0.7-1.3 mg/dL
Glucose	107	70-99 mg/dL
Liver panel		
Albumin	1.9	3.5-5.0 g/dL
Total bilirubin	8.2	0.1-1.2 mg/dL
Alkaline phosphatase	119	40-129 U/L
AST (SGOT)	138	10-40 U/L
ALT (SGPT)	39	7-56 U/L
eGFR	13	>60 mL/min/1.73m^2
Phosphorus	13.6	2.5-4.5 mg/dL

The diagnosis of sepsis was based on clinical evaluation, considering the rapid progression of symptoms after a history of a cat scratch, coupled with laboratory findings indicative of systemic infection. Unfortunately, the patient's condition deteriorated further, culminating in ventricular tachycardia and subsequent cardiac arrest.

It's crucial to highlight the significant impact of the patient's cirrhosis and his compromised immune system on this tragic outcome. While acknowledging the complexity of the patient's condition and possible contributions of multiple comorbidities, it's important to emphasize the importance of considering underlying immunocompromise in cirrhotic patients when investigating infectious causes such as *Bartonella* species. 

## Discussion

In alcoholic liver cirrhosis, the resistance of the immune system to pathogens is greatly weakened. Long-term alcohol consumption weakens the immune system, causing oxidative stress and inflammatory reactions, which in turn lead to the development of liver fibrosis and eventually cirrhosis [5,6]. This progression results in immunosuppression and disruption of the liver and regular immune processes. The ability of neutrophils and macrophages to protect against bacterial infections is significantly reduced in cirrhotic patients. These individuals’ neutrophils show abnormal phagocytosis, intracellular killing, and chemotaxis. Similarly, macrophages exhibit reduced phagocytic activity and altered cytokine production [7]. Hence, the cell’s compromised functionality and altered ability to recognize, engulf, and eliminate pathogens is a major factor in the cirrhotic patient's increased susceptibility to infection. The altered functions of neutrophils and macrophages are further exacerbated by a dysregulated cytokine environment. When produced persistently, elevated levels of interleukin-6 (IL-6) in cirrhotic patients contribute to ongoing inflammation and tissue damage such as liver fibrosis [8,9]. However, its role in host defense against infections means that its dysregulated production can modulate immune responses in complex ways, potentially affecting the body's ability to effectively fight infections. Interleukin-10 (IL-10), a cytokine with anti-inflammatory qualities, is essential for controlling the immune system's reaction to avoid tissue damage. Increased IL-10 production in the setting of cirrhosis can inhibit efficient immune responses and raise the risk of bacterial infections [10,11]. Increased levels of IL-10 in cirrhotic patients inhibit pro-inflammatory cytokine production and antigen-presenting cell activity, perhaps hindering the removal of infections such as CSD-causing agent *B. henselae*.

One of the hallmark responses to *B. henselae* infection is a pronounced vascular proliferation, leading to angiogenesis particularly visible in conditions like bacillary angiomatosis [2]. *B. henselae* stimulates the production of angiogenic factors, like vascular endothelial growth factor, which promotes abnormal vascular proliferation seen in conditions like bacillary angiomatosis. This results in lesion formation primarily in the skin, liver, and spleen. In addition to vascular effects, *B. henselae* can cause regional lymphadenopathy, characterized by swollen, painful lymph nodes near the site of the initial infection. This results from cytokine response to the bacterium, which is characterized by the elevation of inflammatory cytokines such as interleukin-1 and tumor necrosis factor-alpha, that contribute to the inflammation and pain at the site of infection. These cytokines, along with interleukin-8, play a role in the recruitment of immune cells to the site of infection, leading to the characteristic lymphadenopathy.

Given these complexities, the management and diagnosis of infections such as CSD in cirrhotic patients require a nuanced approach. The choice and dosing of antibiotics need to account for the altered metabolism and excretion in the setting of liver dysfunction. Current guidelines suggest that adult patients with lymphadenitis without other signs of CSD should receive a five-day course of azithromycin (500 mg orally on day 1, followed by 250 mg orally daily for four days) [12]. For patients with disseminated disease such as Hepatosplenic disease and fever of unknown origin, the currently recommended antibiotic regimen is rifampin (300 mg orally or IV twice daily) and azithromycin (500 mg orally or IV on day 1, followed by 250 mg orally or IV daily thereafter) [13]. More so, for patients exhibiting neurologic symptoms of CSD, a preferred regimen includes doxycycline (100 mg orally twice daily) and rifampin (300 mg orally twice daily) for four to six weeks [14]. Moreover, the immunosuppressive state of these patients may necessitate prolonged or more aggressive antibiotic therapy to achieve effective pathogen clearance. Our case report patient illustrates that *B. henselae* is challenging to detect and can mimic other conditions, making appropriate treatment difficult. While the patient presented with a cat scratch, lymphadenopathy, fever, and signs of sepsis, the lack of a published connection between the immunocompromised state of cirrhotic patients and severe CSD outcomes led to the administration of broad-spectrum antibiotics to cover other potential etiologies of the presentation.

The accurate diagnosis of CSD in patients with alcoholic liver cirrhosis is critical due to their heightened risk for severe outcomes. The disease's atypical presentation, often mimicking other conditions complicates early identification. Traditional diagnostic criteria for CSD, such as a history of cat scratch, the presence of lymphadenopathy, and possibly a positive skin test, can often be misleading or insufficient. In immunocompromised patients, these clinical signs may be attenuated or present atypically due to their impaired immune response. Consequently, reliance on these criteria alone can lead to diagnostic delays with potentially fatal outcomes. The development of PCR tests and other molecular diagnostic techniques has transformed the way that CSD is identified. Compared to serological testing, PCR testing offers a more accurate and sensitive method for directly identifying *B. henselae* DNA in clinical samples [15]. Due to issues such as immune system differences and cross-reactivity, serological testing may be less accurate. This approach is especially helpful for liver cirrhosis patients, who might not produce enough antibody response or might not respond right away.

We believe that even if CSD had been recognized earlier, it would not have altered the outcome due to the patient's immunocompromised status. While existing literature acknowledges the potential for devastating outcomes in immunocompromised individuals with CSD, it does not specifically address how cirrhosis-induced immunocompromise may exacerbate these outcomes. This underscores the need for clinicians to stay informed about such advancements, especially in managing immunocompromised patients where delays in diagnosis can have fatal consequences.

## Conclusions

The case exemplifies the swift progression of infection to sepsis in patients with liver cirrhosis. The compromised immune system and impaired hepatic function contribute to the vulnerability of these patients. Cat scratches, seemingly trivial, can act as portals for severe infections. In this case, the rapid progression of sepsis prevented effective intervention, even in the presence of maximal medical support. It is imperative for healthcare providers to always remain vigilant for infections in immunocompromised patients, being aware of the significance of prompt diagnosis and the timely initiation of antibiotic treatment. This vigilance highlights the importance of interdisciplinary communication, particularly between infectious disease specialists, hepatologists, and critical care teams, in delivering comprehensive patient care.
